# *Roseburia intestinalis*: A Beneficial Gut Organism From the Discoveries in Genus and Species

**DOI:** 10.3389/fcimb.2021.757718

**Published:** 2021-11-22

**Authors:** Kai Nie, Kejia Ma, Weiwei Luo, Zhaohua Shen, Zhenyu Yang, Mengwei Xiao, Ting Tong, Yuanyuan Yang, Xiaoyan Wang

**Affiliations:** ^1^ Department of Gastroenterology, The Third Xiangya Hospital, Central South University, Changsha, China; ^2^ Hunan Key Laboratory of Nonresolving Inflammation and Cancer, Cancer Research Institute, Central South University, Changsha, China

**Keywords:** *Roseburia*, *Roseburia intestinalis*, probiotic, inflammatory bowel disease (IBD), microbiome

## Abstract

*Roseburia intestinalis* is an anaerobic, Gram-positive, slightly curved rod-shaped flagellated bacterium that produces butyrate in the colon. *R. intestinalis* has been shown to prevent intestinal inflammation and maintain energy homeostasis by producing metabolites. Evidence shows that this bacterium contributes to various diseases, such as inflammatory bowel disease, type 2 diabetes mellitus, antiphospholipid syndrome, and atherosclerosis. This review reveals the potential therapeutic role of *R. intestinalis* in human diseases. Patients with inflammatory bowel disease exhibit significant changes in *R. intestinalis* abundance, and they may benefit a lot from modulations targeting *R. intestinalis*. The data reviewed here demonstrate that *R. intestinalis* plays its role in regulating barrier homeostasis, immune cells, and cytokine release through its metabolite butyrate, flagellin and other. Recent advancements in the application of primary culture technology, culture omics, single-cell sequencing, and metabonomics technology have improved research on *Roseburia* and revealed the benefits of this bacterium in human health and disease treatment.

## Introduction

*Roseburia* spp. have been brought to the fore because of their novel role in modulating the gut microbial ecology, immune responses, and the development of human disorders (Tamanai-Shacoori et al., 2017). *Roseburia* was named in honor of Theodor Rosebury, an American microbiologist, for his groundbreaking contributions to the field of oral microbiome. *Roseburia* sp. belongs to the phylum Firmicutes, class Clostridia, order Clostridiales, and family Lachnospiraceae (Stackebrandt, 2014). The *Roseburia* genus has five well-characterized species (*Roseburia intestinalis*, *Roseburia hominis*, *Roseburia inulinivorans*, *Roseburia faecis*, and *Roseburia cecicola*), all of which produce short-chain fatty acids (SCFAs), such as acetate, propionate, and butyrate (Tamanai-Shacoori et al., 2017). In 2002, Duncan et al. isolated a strain of anaerobic, Gram-positive, slightly curved rod-shaped, and flagellated bacteria from human fecal samples. Subsequent phylogenetic analysis revealed its intimate similarity with *R. cecicola* and the members of cluster XIVa of *Clostridium subphylum*; thus, it was named *Roseburia intestinalis* (Duncan et al., 2002). Among the top 20 most abundant bacteria in the gut microbiome, the *R. intestinalis* cluster usually accounts for 0.9%–5.0% (mean = 2.3%) of the total microbiota (Hold et al., 2003; Hiippala et al., 2018).

*R. intestinalis* are obligate anaerobes that are difficult to culture. They were firstly isolated from human feces in an M2 medium-based culture system (Louis et al., 2004). It was reported that *R. intestinalis* XB6B4 can be cultivated under anaerobic conditions at 37°C in a complex medium containing clarified rumen fluid (described in Chassard et al., 2007) and 0%–5% complex substrates (oat spelt xylan, wheat or corn bran, pea fiber, cabbage, and leek) or 0%–3% sugar (xylose or glucose) (Chassard et al., 2007; Mirande et al., 2010). *R. intestinalis* DSM 14610 was reported to degrade and utilize oligofructose as the sole energy source, but only when acetate was added to a special medium for colon bacteria (Falony et al., 2006). This bacterium can also grow anaerobically at 37°C in lytic/10 anaerobic/F medium or LYBHI medium supplemented with yeast extract, cellobiose, maltose, and cysteine (Zhu, C. et al., 2018; Cornuault et al., 2020). Butyryl-CoA:acetate CoA transferase is the key enzyme that produces butyrate in butyrate-producing bacteria. This enzyme has been detected in *R. intestinalis*, which endows it with the ability to utilize acetate in order to produce butyrate (Pryde et al., 2002). Previous studies have found that *R. intestinalis* and *Faecalibacterium prausnitzii* are the most abundant butyrate-producing bacteria in human feces (Hold et al., 2003; Duncan et al., 2004). *R. intestinalis* can ferment fibers to produce butyrate in an M2 medium supplemented with wheat starch (Louis et al., 2004). Of note is that butyrate has been reported to exert pervasive anti-inflammatory and metabolic modulation effects in different disease models (Canfora et al., 2015; McNabney and Henagan, 2017). Therefore, *R. intestinalis* can be applied as a potential probiotic given its ability to produce butyrate. Enteric bacteria use fibers as a source of carbon to produce butyrate. Martinez et al. reported that a whole-grain diet increases the intestinal abundance of *R. intestinalis* and improves the concentration of interleukin-6 (IL-6, which is linked to metabolic dysfunctions) (Martinez et al., 2013). *R. intestinalis* also has the ability to ferment xylan and β-mannan, which are common fiber ingredients in the diet (Mirande et al., 2010; Leth et al., 2018; La Rosa et al., 2019). β-mannans and xylans are important components of the plant cell wall, and they are acetylated to protect them from degradation by glycoside hydrolases. β-mannans are widely present in human and animal diets. RiCE2 and RiCE17, two carbohydrate esterases from *R. intestinalis*, remove acetylations from all positions in complex β-mannans in a complementary manner (Michalak et al., 2020). The growth of *R. intestinalis* is influenced by the carbon source, symbiont, and anoxia, and also by the pH. It has been discovered that iron can modulate the ability of *R. intestinalis* to produce butyrate through the pyruvate mechanism: ferredoxin oxidoreductase (PFO) can convert pyruvate to acetyl-CoA (Dostal et al., 2015). In addition, PFO is sensitive to reducing ferredoxin (Dostal et al., 2015). These data show that *R. intestinalis* uses different carbohydrates as the source of energy and that its growth is susceptible to iron. The human regenerating family member 3 alpha (hREG3α) promotes the growth of *R. intestinalis* by inhibiting reactive oxygen species (ROS) signaling and induces resistance in mice with dextran sulfate sodium (DSS)-induced colitis (Darnaud et al., 2018). Colonic bacteria compete for the colonic ecological niche through nutrition plunder and preempt niche advantage (Leth et al., 2020). Several external factors, such as diet or antibiotic use, and endogenous factors may affect the ecological niche of *R. intestinalis*. It has been reported that ultravirulent phage mutants can drive the disruption of *R. intestinalis* and the ecological niche occupied by other resistant bacteria (Cornuault et al., 2020). This phenomenon reveals that the interactions among bacteria in the gut environment are complex. The structural characteristic of the flagella in *R. intestinalis* regulates its motion. The flagella help *R. intestinalis* to penetrate the colonic mucus layer in order to interact with the epithelium. Therefore, *R. intestinalis* is one of the most abundant butyrate-producing bacteria that adhere to intestinal mucin, facilitating its significant advantages for the probiotic role (Van den Abbeele et al., 2013).

**Figure 1:**
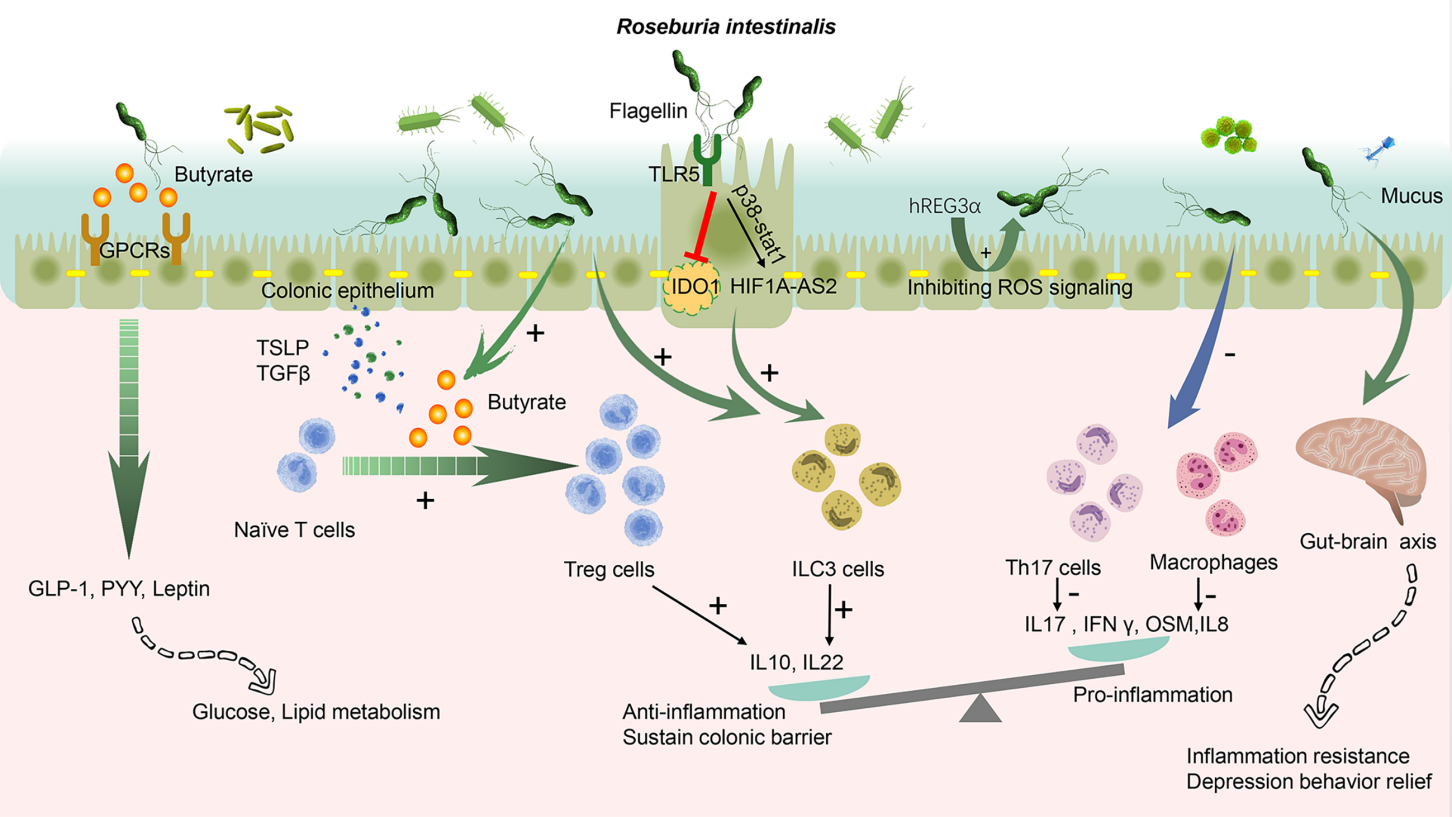
*Roseburia intestinalis* modulation in the colonic tract. The butyrate produced *by R. intestinalis* exerts an extensive effect on energy metabolism, gut barrier, and anti-inflammation. *R. intestinalis* stimulates enteric cells, thereby excreting cytokines, promoting the differentiation of regulatory T cells (Tregs), and activating type 3 innate lymphoid cells (ILC3). It also suppresses Th17 and macrophages. Its flagellin displays an anti-inflammation effect through TLR5. The biological effect induced by *R. intestinalis* exhibits a significant probiotic-like role. *R. intestinalis* also influences the energy metabolism and the gut–brain axis. A *plus sign* indicates promote and a *minus sign* indicates inhibit. *GPCRs*, G-protein coupled receptors; *TSLP*, thymic stromal lymphopoietin; *GLP-1*, glucagon-like peptide-1; *PYY*, peptide YY; *OSM*, oncostatin M; *hREG3α*, human regenerating family member 3 alpha; *TLR5*, Toll-like receptor 5; *IDO1*, indoleamine 2,3-dioxygenase-1; *HIF1A-AS2*, lncRNA (HIF1A-AS2).

**Figure 2:**
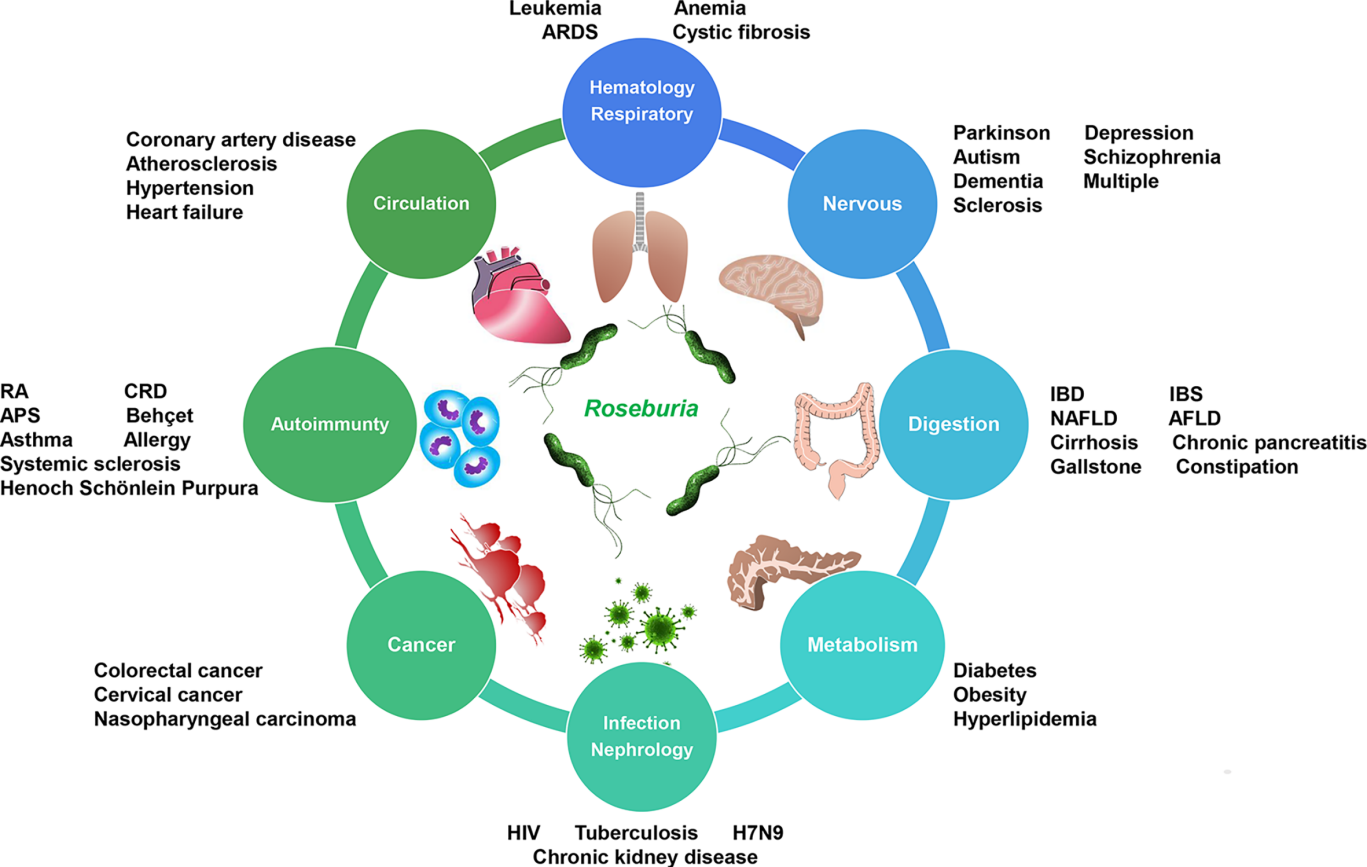
*Roseburia* dysbiosis-associated diseases exist in different systems. *IBD*, inflammatory bowel diseases; *IBS*, irritable bowel syndrome; *NAFLD*, non-alcoholic fatty liver disease; *AFLD*, alcoholic fatty liver disease; *CRD*, chronic rheumatoid disease; *APS*, antiphospholipid syndrome; *HIV*, human immunodeficiency virus; *H7N9*, avian influenza A (H7N9); *ARDS*, acute respiratory distress syndrome.

According to the guidelines from the World Health Organization (WHO) on the Evaluation of Probiotics in Food issued in 2002, probiotics are viable microorganisms in dietary supplements that, when consumed at certain levels, stabilize the gastrointestinal tract microflora and confer health benefits to the consumer. In addition to our attempts to expand knowledge about *R. intestinalis* (**Table 1** and **Figure 1**), multiple trials and experiments based on animal models have elaborated the complicated and tight relationship between *R. intestinalis* and the occurrence of human diseases (**Figure 2**). Accumulating evidence also supports the probiotic effect of *R. intestinalis* in the human body, which demonstrates its fundamental benefits in maintaining a healthy homeostasis. However, the vast majority of the studies described the role of *R. intestinalis* through microbiome and metabolomics analyses (**Table 2**). *R. intestinalis* frequently appears in the list of significantly variable bacteria of different diseases and has been shown to have a beneficial role. Numerous studies have also elucidated the effect of *R. intestinalis* on immunity and pathophysiology. As such, there is a need to clarify the role of *R. intestinalis* within separate systems.

**Table 1:** Prior bacterial discoveries associated with *Roseburia intestinalis*.

Study	Nationality	Year	Journal	Findings
(Duncan et al., 2002)	UK	2002	IJSEM	*Roseburia intestinalis* sp. nov., a novel saccharolytic, butyrate-producing bacterium from human feces.
(Hold et al., 2003)	UK	2003	A.E.M	*Roseburia intestinalis*: 2.3% of fecal microbiota populations at mean. The genus *Roseburia* is among the most abundant known butyrate-producing bacteria in human feces.
(Duncan et al., 2004)	UK	2004	A.E.M	*Roseburia* spp. seem to have a consistent contribution for acetate to butyrate formation.
(Mirande et al., 2010)	France	2009	J Appl Microbiol	*Roseburia intestinalis* is a dominant xylanolytic bacterium in dietary fiber degradation and fermentation.
(Van den Abbeele et al., 2013)	Belgium	2012	ISME J	*Roseburia intestinalis* colonizes mucins most specifically with flagella and may allow for penetration into the mucus layer.
(Martinez et al., 2013)	USA	2012	ISME J	Whole-grain barley enriches *Roseburia intestinalis* and improves metabolic dysfunctions.
(Qin et al., 2012)	China	2012	Nature	*Roseburia intestinalis* is one of the markers that might be useful for classifying type 2 diabetes.
(Russell et al., 2013)	UK	2013	MNFR	*Roseburia intestinalis* can ferment tryptophan indole-3-carboxylic acid.
(Arpaia et al., 2013)	USA	2013	Nature	Butyrate from colonic microorganisms promotes the differentiation of Tregs and influences the pro- and anti-inflammatory balance.
(Dostal et al., 2015)	Switzerland	2015	J Nutr	Iron modulates butyrate production in *Roseburia intestinalis*.
(Hoffmann et al., 2016)	France	2016	ISME J	*Roseburia intestinalis* induced a decreased IFNγ and IL-17 production with increased IL-22 production.
(Hegazy et al., 2017)	UK	2017	Gastroenterology	*Roseburia intestinalis* reactive CD4^+^ T cells support intestinal homeostasis, and their function is altered during inflammation.
(Kasahara et al., 2018)	USA	2018	Nat Microbial	*Roseburia intestinalis* interacts with dietary plant polysaccharides to provide protection against atherosclerosis.
(Shen et al., 2018)	China	2018	JGH	*Roseburia intestinalis* alleviates experimental colitis pathology by inducing anti-inflammatory responses.
(Quan et al., 2018)	China	2018	BBRC	*Roseburia intestinalis*-derived flagellin is a negative regulator of intestinal inflammation.
(Zhu C. et al., 2018)	China	2018	Mol Med Rep	*Roseburia intestinalis* inhibits interleukin-17 excretion and promotes regulatory T-cell differentiation.
(Seo et al., 2020)	Korea	2019	Cell Host Microbe	*Roseburia intestinalis* improves the gut ecosystem, leading to ameliorated alcoholic fatty livers.
(Tan et al., 2019)	China	2019	Scandinavian J.G	*Roseburia intestinalis* inhibits oncostatin M and maintains tight junction integrity in a murine model.
(Luo et al., 2019)	China	2019	Mol Med Rep	*Roseburia intestinalis* supernatant ameliorates colitis induced in mice by regulating the immune response.
(La Rosa et al., 2019)	Norway	2019	Nat Com	*Roseburia intestinalis* is a primary degrader of dietary β-mannans.
(Ruff et al., 2019)	USA	2019	Cell Host Microbe	*Roseburia intestinalis* cross-reacts with T and B cells to induce auto-APS antibody.
(Kellermayer, 2019)	USA	2019	Gastroenterology	*Roseburia* species: prime candidates for microbial therapeutics in inflammatory bowel disease.
(Xu et al., 2021)	China	2021	Therap Adv Gastroenterol	*Roseburia intestinalis* modulates the gut homeostasis by the gut–brain axis.
(Montalban-Arques et al., 2021)	Switzerland	2021	Cell Host Microbe	*Roseburia intestinalis* exerts synergic therapeutical effects in anti-PD-1 therapy of colorectal cancer.

**Table 2:** Studies associated with *Roseburia* spp. in different diseases of the different systems.

Study	Country	System	Disease	Source	Size	Sample	Method	Findings
(Gevers et al., 2014)	USA.	Digestion	Crohn’s	Human	668	Feces	16S rRNA	*Roseburia intestinalis* **↓**
(Morgan et al., 2012)	USA.	Digestion	Crohn’s	Human	223	Feces	16S rDNA V3–V5	*Roseburia* ↓
(Kumari et al., 2013)	India	Digestion	UC	Human	40	Feces	FISH–flow cytometry	*Roseburia* ↓
(Rajilic-Stojanovic et al., 2013)	Serbia	Digestion	UC	Human	45	Feces	16S rRNA	*Roseburia* spp. ↓
(Chassard et al., 2012)	France	Digestion	IBS	Human	26	Feces	FISH	*Roseburia* ↓
(Gobert et al., 2016)	France	Digestion	IBS	Human	91	Feces	16S rRNA V5–V6	*Roseburia* ↓
(Parthasarathy et al., 2016)	USA.	Digestion	Constipation	Female	50	Mucosa feces	16S rRNA V3–V5	*Roseburia* correlated with faster colonic transit
(Zhu et al., 2013)	USA.	Digestion	NAFLD	Human	53	Feces	16S rRNA	*Roseburia* ↓
(Saltzman et al., 2018)	Australia	Digestion	NAFLD	Human			Review	*Roseburia* ↓
(Raman et al., 2013)	UK	Digestion	NAFLD	Human	60	Feces	16S rRNA	*Roseburia* ↑
(Seo et al., 2020)	Korea	Digestion	AFLD	Human	212	Feces	16S rRNA	*Roseburia* spp. ↓
(Bajaj et al., 2018)	USA.	Digestion	Cirrhosis	Human	180	Feces	16S rRNA, 16S rDNA	*Roseburia* was protective against hospitalizations in the DNA model
(Bajaj et al., 2012)	USA.	Digestion	Cirrhosis	Human	53	Mucosa	16S rRNA	*Roseburia* ↓ in hepatic encephalopathy mucosal microbiome
(Forbes et al., 2018)	Canada	Autoimmunity	Rheumatoid arthritis	Human	44	Feces	16S rRNA V4	*Roseburia* ↓
(Consolandi et al., 2015)	Italy	Autoimmunity	Behçet syndrome	Human	38	Feces	16S rRNA	*Roseburia* ↓
(Patrone et al., 2017)	Italy	Autoimmunity	Systemic sclerosis	Human	18	Feces	16S rRNA V3–V4	*Roseburia* ↓ in patient with gastrointestinal involvement
(Salem et al., 2019)	France	Autoimmunity	CRD	Human	1084	Feces	Review	*Roseburia* ↓
(Ruff et al., 2019)	USA.	Autoimmunity	APS	Human	35	Feces	16S rRNA V4	*Roseburia intestinalis* involved APS occuring
(Qin et al., 2012)	China	Metabolism	Diabetes	Human	345	Feces	MGWAS	*Roseburia intestinalis* ↓
(Tilg and Moschen, 2014)	Australia	Metabolism	Diabetes	Human			Review	*Roseburia intestinalis* ↓
(Leiva-Gea et al., 2018)	Spain	Metabolism	Diabetes	Human	43	Feces	16S rRNA V2–V3	*Roseburia* ↓
(Jamshidi et al., 2019)	Iran	Metabolism	Diabetes				Review	The most common bacterial alterations included *Roseburia* spp.
(Tims et al., 2013)	Netherlands	Metabolism	Obesity	Human	80	Feces	16S rRNA	*Roseburia intestinalis* ↑
(Gargari et al., 2018)	Italy	Metabolism	Hyperlipidemia	Human	30	Feces	16S rRNA	*Roseburia* ↓
(Keshavarzian et al., 2015)	USA.	Nervous	Parkinson’s	Human	72	Feces	16S rRNA V4	*Roseburia* ↓
(Sampson et al., 2016)	USA.	Nervous	Parkinson’s	Human	12	Feces	16S rRNA V4	*Roseburia* spp. ↑
(Zheng et al., 2016)	China	Nervous	Depression	Human	121	Feces	16S rRNA V4–V5	*Roseburia* ↓
(Jiang et al., 2015)	China	Nervous	Depression	Human	76	Feces	16S rRNA V3–V4	Roseburia ↑
(Liu et al., 2016)	China	Nervous	Depression	Human	100	Feces	16S rRNA	*Roseburia* as a feature of depression compared with D-IBS
(Karlsson et al., 2012)	Sweden	Circulation	Atherosclerosis	Human	25	Feces	Sequencing	*Roseburia* ↓
(Kasahara et al., 2018)	USA.	Circulation	Atherogenesis	Mice	342	Ceca	16S rRNA	*Roseburia intestinalis* ↓
(Liu et al., 2019)	China	Circulation	CAD	Human	201	Feces	16S rRNA V3–V4	Bacterial co-abundance group at different stages of CAD was represented by *Roseburia.*
(Zhu Q. et al., 2018)	China	Circulation	CAD	Human	168	Feces	16S rRNA V4	*Roseburia* ↓
(Yan et al., 2017)	China	Circulation	Hypertension	Human	120	Feces	MGWAS	*Roseburia* spp. ↓
(Qin et al., 2018)	China	Circulation	Hypertension	Rat	16	Feces	16S rRNA	*Roseburia* ↓
(Zheng et al., 2019)	China	Circulation	Heart failure	Rat	30	Feces	Sequencing	*Roseburia* ↓ in the 4w-HF group
(Rajagopala et al., 2016)	USA.	Hematology	Leukemia	Human	51	Feces	16S rRNA	*Roseburia* ↓
(Bindels et al., 2015)	Belgium	Hematology	Leukemia	Mice	33	Feces	16S rRNA	Increased *Roseburia* spp. ameliorated leukemia progression.
(McClorry et al., 2018)	Peru	Hematology	Anemia	Infant	95	Feces	16S rRNA V4	*Roseburia* ↓
(Dostal et al., 2012)	Switzerland	Hematology	Anemia	Rat	40	Feces	16S rRNA V2–V3	*Roseburia* spp. ↓
(Dillon et al., 2017)	USA.	Infection	HIV	Human	32	Mucosa	16S rRNA	*Roseburia intestinalis* ↓
(McHardy et al., 2013)	USA.	Infection	HIV	Human	60	Mucosa	16S rRNA V4	*Roseburia* ↓ in those not receiving anti-retroviral therapy.
(Guillen et al., 2019)	Spain	Infection	HIV	Human	156	Feces	16S rRNA	*Roseburia intestinalis* predicts the presence of gut dysbiosis.
(Maji et al., 2018)	India	Infection	Tuberculosis	Human	12	Feces	16S rRNA V3	*Roseburia* ↑
(Hu et al., 2019)	China	Infection	Tuberculosis	Human	61	Feces	Sequencing	*Roseburia* ↓
(Liang et al., 2017)	China	Cancer	CRC	Human	439	Feces	16S rRNA	*Roseburia intestinalis* ↓
(Yu et al., 2017)	China	Cancer	CRC	Human	168	Feces	MGWAS	*Roseburia intestinalis* ↓
(Chen et al., 2013)	China	Cancer	CRC	Human	100	Feces	16S rRNA V1–V3	*Roseburia* ↓
(Wang et al., 2012)	China	Cancer	CRC	Human	94	Feces	16S rRNA V3	*Roseburia* ↓
(Jiang et al., 2019)	China	Cancer	Nasopharyngeal carcinoma	Human	59	Feces	16S rRNA V3–V4	*Roseburia* spp. ↓
(Wang et al., 2019)	China	Cancer	Cervical cancer	Human	13	Feces	16S rRNA V4	*Roseburia* spp. were more abundant in the CCa group.
(Li et al., 2014)	China	Respiration	ARDS	Rat	16	Feces	16S rRNA V4	*Roseburia* ↓
(Xu et al., 2017)	China	Urinary	CKD	Human	64	Feces	16S rRNA V4	*Roseburia* ↓
(Jiang et al., 2017)	China	Urinary	CKD	Human	112	Feces	qPCR, 16S rRNA	*Roseburia* ↓
(Wu, I. W. et al., 2020)	China	Urinary	CKD	Human	130	Feces	16S rRNA V4	*Roseburia* was negatively correlated with CKD severity.

Arrows ↑ ↓ : the Roseburia changes in specific disease patients compared with controls.

UC, ulcerative colitis; IBS, irritable bowel syndrome; FISH, fluorescence in situ hybridization; MGWAS, metagenome-wide association study; NAFLD, non-alcoholic fatty liver disease; AFLD, alcoholic fatty liver disease; CRD, chronic rheumatoid disease; APS, antiphospholipid syndrome; CAD, coronary artery disease; HIV, human immunodeficiency virus; H7N9, avian influenza A (H7N9); CRC, colorectal cancer; ARDS, acute respiratory distress syndrome; NMOSD, neuromyelitis optical spectrum disorders.

## Digestive System

### The Role of *R. intestinalis* in Digestive Diseases

*R. intestinalis*, as a digestive tract resident, plays a role in metabolic reprogramming, immune activation, and in sustaining the gut barrier. Notable prominent features of inflammatory bowel disease (IBD) [encompassing Crohn’s disease (CD) and ulcerative colitis (UC)] include microbiota dysbiosis, impaired innate immunity, and extensive tissue damage (mainly in the colon). Global cohorts (**Table 2**) have uncovered the dysbiosis microbiome profile of IBD with significantly low numbers of *Roseburia*. A large sample size-based cohort study of the microbiome research project involving 668 CD patients and healthy subjects revealed a low number of *R. intestinalis* in CD patients (Gevers et al., 2014). Functional enrichment analysis further demonstrated more oxidative stress-activated signaling pathways and fewer carbohydrate and amino acid metabolism pathways associated with nutrition transportation and absorption (Gevers et al., 2014). Another pediatric CD study reported a low abundance of core bacteria, including *Faecalibacterium* and *Roseburia*, in patients with ileal CD (Morgan et al., 2012). Antibiotic exposure was demonstrated to magnify the above-mentioned intestinal dysbiosis in CD patients. Previous investigations by our research group revealed similar results of reduced *Roseburia* in CD patients (Shen et al., 2018). UC and CD have similar changes in *Roseburia*, although their signatures in genetics and pathophysiology are widely different. For instance, Kumari revealed lower abundance of *R. intestinalis* and decreased levels of fecal SCFAs in UC patients compared to the healthy group, particularly for butyrate (Kumari et al., 2013). Elsewhere, a study conducted in Serbia involving 15 UC patients and 15 healthy controls reported decreased levels of *Roseburia* sp. in the microbiota of UC patients, and the dysbiosis in UC was persistent in time and segment dimensions (Rajilic-Stojanovic et al., 2013). Therefore, it is increasingly becoming apparent that *Roseburia* is depleted in IBD *via* a mechanism associated with the anti-inflammatory effect, barrier protective effect, and the close connection of *R. intestinalis* with innate immunity. Irritable bowel syndrome (IBS) is a colonic transit and sensory disorder characterized by constipation, diarrhea, or mixed subgroups. Results from 16S ribosomal RNA (rRNA) sequencing and fluorescence *in situ* hybridization (FISH) analysis in a French cohort showed that constipated-IBS (C-IBS) patients had lesser *Roseburia* abundance than did the controls (Chassard et al., 2012; Gobert et al., 2016). These findings demonstrate that both IBS and constipation are likely associated with low *Roseburia* abundance and impaired colonic transit. For instance, a previous investigation of the colonic mucosal microbiota, fecal microbiota, and the transit signature between constipated patients and healthy controls revealed a correlation of the *Roseburia* genus with faster colonic transit (Parthasarathy et al., 2016). Butyrate, mainly metabolized in the liver, exerts a local inhibitory effect against liver adipogenesis and adipose accumulation (Canfora et al., 2015). A high-fat supplement elevates the triglyceride content and decreases β-oxidation within the liver. Studies have demonstrated that butyrate or butyrate-producing bacterial strains are related with reduced ectopic lipids in a non-alcoholic fatty liver disease (NAFLD) animal model (Mattace Raso et al., 2013; Jin et al., 2015). Based on existing factors, there is strong evidence that *Roseburia* is more underrepresented in NAFLD patients than in the control group (Zhu et al., 2013; Saltzman et al., 2018). However, some researchers reported divergent results and proposed that the altered diet and habitat effects may explain the discrepancy in the results (Raman et al., 2013). Seo et al. (2020) reported that alcoholic fatty liver disease (AFLD) displayed a significant depletion of *R. intestinalis*, and such dysbiosis correlates with a medical history of alcohol intake in a cohort of Korean twins (Seo et al., 2020). *R. intestinalis* potentially ameliorated the experimental AFLD model by restoring the gut barrier integrity. The flagellin of *R. intestinalis* is an active microbial ingredient that elevates tight junction protein and modulates immune response *via* the TLR5 receptor (Seo et al., 2020). In other digestive disorders such as cirrhosis, the fecal microbial sequencing results of cirrhosis patients showed that *Roseburia* protected against hospitalization. This study used fecal RNA instead of DNA for microbial 16S rRNA sequencing in order to better understand the bacterial metabolic activity (Bajaj et al., 2018). Hepatic encephalopathy is a severe complication of hepatic cirrhosis, commonly accompanied by impaired gut permeability. In a past study, lower *Roseburia* abundance was reported in sigmoid mucosal microbiota (Bajaj et al., 2012). Of note is that the majority of studies support the view that *Roseburia* exerts a protective role in most digestive diseases. *R. intestinalis*, together with the metagenome, plays beneficial functions in maintaining health and preventing diseases in animal and cell models. However, the primary culture of *R. intestinalis* strains remains a major hindrance, and there is a lack of well-designed clinical trials to confirm the protective role of *R. intestinalis*.

### Mechanisms of *R. intestinalis* in Digestive Diseases

Extensive effects of *R. intestinalis* and its derivatives have been implicated in immune modulation and inflammatory regulation. *R. intestinalis* primarily functions as a primary butyrate-producing bacterium. Butyrate produced by commensal microbes was revealed to promote the proliferation of extrathymic regulatory T cells (Tregs) through intrinsic enhancer conserved non-coding sequence 1 (CNS1) (Arpaia et al., 2013). The mechanism was essential for extrathymic Treg differentiation, but dispensable for thymic Tregs. Tregs, as vital anti-inflammatory lymphocytes, produce interleukin-10 (IL-10), transforming growth factor beta (TGF-β), and interferon gamma (IFNγ). Microbial butyrate has been demonstrated to contribute to inner pro- and anti-inflammatory balance by influencing the differentiation of Tregs (Hegazy et al., 2017). In view of these findings, our research group investigated the role of Tregs in mouse models induced by bacterial suspension gavage and cell line co-culture. The results demonstrated that *R. intestinalis* inhibited the secretion of interleukin-17 (IL-17) and promoted the differentiation of Tregs in 2,4,6-trinitrobenzene sulfonic acid (TNBS)-induced colitis (Zhu C. et al., 2018). Recent evidence showed that 5-hydroxyindoleacetic acid (5-HIAA) produced *via* butyrate stimulation can bind the aryl hydrocarbon receptor (AhR) in regulatory B cells (Bregs), inducing suppressive effects (Michaudel and Sokol, 2020). In memory T cells, butyrate activates β-oxidation and significantly influences the state and function of cells (Bachem et al., 2019). It is possible that an impaired production of butyrate may contribute to the pro-inflammatory polarization of intestinal macrophages. In addition, butyrate potentially regulates the intestinal macrophage function *via* histone deacetylase inhibition, and this causes a dysfunction of the immune response (Michaudel and Sokol, 2020). Evidence shows that gut microbiota-derived butyrate modulates the functions of type 2 innate lymphoid cells (ILC2), which block their uncontrolled activation; as such, they consequently exert a negative role in lung inflammation and asthma (Lewis et al., 2019). Notably, the involvement of intracellular metabolism is supported by the butyrate-mediated induction of changes in mitochondrial ROS (mROS) production and glycolysis (Lewis et al., 2019). Additionally, the preferential use of Fas over glucose by ILC2 to maintain their function in infection or nutritional stress suggests a direct role of butyrate in fueling the tricarboxylic acid cycle (Michaudel and Sokol, 2020). Consequently, butyrate derived from *R. intestinalis* may exert a profound immune effect in the gut and establish the unique status of *R. intestinalis.*

Together with similar models, our study demonstrated another mechanism of *R. intestinalis* in decreasing the secretion of the macrophage-derived oncostatin M (OSM) and maintaining intestinal mucosal permeability to alleviate DSS-induced colitis (Tan et al., 2019). We elucidated Crohn’s bacterial composition signature with reduced *R. intestinalis* in CD through 16S rRNA sequencing of fecal samples obtained from CD patients and healthy controls (Yang et al., 2020). In a subsequent experiment, we discovered that *R. intestinalis* alleviates intestinal inflammation by enhancing the proliferation of Tregs and promoting the secretion of anti-inflammatory cytokines [IL-10, TGF-β, and thymic stromal lymphopoietin (TSLP)] (Shen et al., 2018). Through gas chromatography–mass spectrometry (GC-MC) analysis, we affirmed that SCFAs were present in the *R. intestinalis* non-protein supernatant. Elsewhere, the *R. intestinalis* non-protein supernatant was found to alleviate TNBS- and DSS-induced colitis by reducing the percentages of colonic inflammatory macrophages and Th17 cells. Such shifts downregulated the IL-6 and STAT3 in both mouse models and cell lines (Luo et al., 2019). The above-described findings suggest a significant anti-inflammatory and immune-modulating effect of the butyrate produced by *R. intestinalis* exhibited in the gut. Our novel findings are consistent with the results reported by (Arpaia et al., 2013 and Hegazy et al., 2017).

A previous investigation by (Patterson et al., 2017) demonstrated that *R. hominis* potentially strengthens the function of the gut barrier and enhances the expansion of the population of Tregs, possibly *via* flagellin/Toll-like receptor 5 (TLR5) signaling. Also, (Vijayan et al., 2018) found that activated TLR5 could stimulate type 3 innate lymphoid cells (ILC3) and promote the production of interleukin-22 (IL-22), which sustains the gut barrier function. In our research, for a clear definition of the physiological role of flagellin, we used the recombinant flagellin of *R. intestinalis*. The analysis revealed that flagellin played an inflammatory inhibitory role by upregulating lncRNA (HIF1A-AS2) *via* p38/STAT1 signaling, exhibiting an anti-inflammatory role (Quan et al., 2018). At the same time, *R. intestinalis*-derived flagellin ameliorated colitis by targeting the miR-223-3p-mediated activation of NLRP3 inflammasome and pyroptosis. The method combined miRDB and TargetScan database prediction and experiment validation in order to ascertain the beneficial role of *R. intestinalis* in colitis (Wu, I. W. et al., 2020). ILC3s is a member of innate lymphatic cells, and its widespread effects in pathogen resistance, regulation of autoimmune inflammation, tissue remodeling, cancer, and metabolic homeostasis have been reported (Klose and Artis, 2016). *R. intestinalis* can ferment tryptophan into indole-3-carboxylic acid, a member of the indoles (Russell et al., 2013). In addition, some tryptophan metabolites (e.g., indoles including indole-3-carboxylic acid) may activate the AhR of ILC3. For instance, Hoffmann et al. (2016), using gnotobiotic mice transplanted with *R*. *intestinalis*, investigated the physiological impact on the host from the aspects of colonic histology, SCFAs, immune signature, transcriptome, and bile acid metabolism. They revealed that *R. intestinalis* could induce the upregulation of IL-22 and the downregulation of IL-17 and IFNγ in bacteria-transplanted gnotobiotic mice (Hoffmann et al., 2016). Corresponding findings also support an essential role of ILC3 in the immune modulation of *R. intestinalis*. In our recent study, we have evaluated whether *R. intestinalis* could significantly influence the activity of indoleamine 2,3-dioxygenase-1 (IDO1, an enzyme dominating tryptophan metabolism) and compared the results to other glycolysis lipolytic and amino acid metabolic enzymes (unpublished). In another recent project in 2019, we aimed to investigate the balancing mechanism between intracellular and extracellular tryptophan metabolism. The results of the study demonstrated that intracellular IDO1-mediated tryptophan metabolism potentially altered *R. intestinalis* flagellin–TLR5 signaling. Also, extracellular tryptophan metabolic indoles were suggested to activate ILC3 and IL-22 production *via* the AhR. Notably, the combined effect may induce colonic and systemic anti-inflammatory effects by immunometabolism. More and more evidence had accumulated to support the potential role the gut–brain axis plays in digestive disorders. For example, *R. intestinalis* may function in the modulation of the gut–brain axis by reducing the level of colonic 5-hydroxytryptamine (5-HT), inhibiting the expression of glial fibrillary acidic protein (GFAP), and alleviating the depression-like behaviors in colitis model mice (Xu et al., 2021). These pieces of evidence provide a new perspective on the therapeutic role of *R. intestinalis* in inflammatory bowel diseases.

## Autoimmune Disorders

Autoimmune diseases usually involve multiple systems, including the intestine. Bacteria interact with enteric mucosal immune cells by presenting antigen and chemical signals to organs, such as the thymus, brain, liver, and the pancreas. It is universally accepted that the immune system mounts a response to invaders. Usually, autoimmune diseases have complicated pathogenesis associated with gut microbes. According to the sequencing results of the fecal samples obtained from 44 Canadian rheumatic arthritis (RA) patients, Forbes et al. found a decreased *Roseburia* abundance, which was used to distinguish RA patients from the healthy group in a random forest classification model (Forbes et al., 2018). In addition, the *Roseburia* genus was a potential marker of health given its butyrate-producing and anti-inflammatory properties (Forbes et al., 2018). Notably, identical lower gut *Roseburia* abundance phenomena were found in adults with Behçet syndrome and systemic sclerosis, particularly in systemic sclerosis patients with gastrointestinal involvement (Consolandi et al., 2015; Patrone et al., 2017). However, these studies were limited by the small sample size of the cohorts. Elsewhere, a systematic literature review of studies involving the collection of fecal samples from 1,084 patients with chronic rheumatic diseases and healthy controls revealed a decreased number of *Roseburia* species in enrolled patients (Salem et al., 2019).

Bacteria sometimes imitate autologous antigens to induce autoimmune antibodies. In antiphospholipid syndrome (APS), researchers have discovered cross-reactivity between non-orthologous mimotopes expressed by *R. intestinalis* and the autoantigen β2-glycoprotein (β2GPI) (Ruff et al., 2019). Besides, the anti-*R. intestinalis* mimotope immunoglobulin G (IgG) was significantly elevated in APS patients and was correlated with anti-β2GPI IgG autoantibodies. Meanwhile, *R. intestinalis* gavage could trigger an autoimmune pathology in susceptible mice (Ruff et al., 2019). However, due to *R. intestinalis* appearing as a type of common gut-friendly bacteria, the specific autoimmune phenotype transformation needs stricter consideration in such a mouse model. Otherwise, the on–off switch may hide in a particular microenvironment. These findings provide strong evidence that *R. intestinalis* displays beneficial effects in most patients with autoimmune diseases despite the occasionally complicated effects. As such, further exploration is warranted to elucidate the mechanism of the interaction of *R. intestinalis* with the immune system, an adaptation of immune cells, perception, and evolution with intestinal microbes.

## Metabolic Diseases

Through dysbiosis-associated Kyoto Encyclopedia of Genes and Genomes (KEGG) enrichment analysis, researchers have demonstrated that intestinal microbiome dysbiosis alters the commensal remolding and shifts the nutritional metabolism. Accumulating evidence reveals the tight association of butyrate with glucose homeostasis, insulin resistance, and appetite (Canfora et al., 2015). Metabolic diseases are typically multi-nutrition-utilizing disorders and abnormalities of immune-induced hormones. As mentioned above, *Roseburia* spp. are a critical butyrate-producing bacteria cluster. Several researchers have reported that the potential role of butyrate is by inhibiting histone deacetylase (HDAC) or interacting with G protein-coupled receptors (GPCRs) such as free fatty acid receptors 2 (FFAR2) and 3 (FFAR3) in the control of body weight and insulin sensitivity (Canfora et al., 2015; Morrison and Preston, 2016; McNabney and Henagan, 2017). Butyrate can increase energy expenditure and fat oxidation, thereby exerting a further influence on energy homeostasis and lipid metabolism. Studies have also reported that FFAR 2/3-associated signaling exerts a potential regulatory role for butyrate in glucose homeostasis (Canfora et al., 2015). To date, there is strong evidence on the relationship between *R. intestinalis* and type 2 diabetes mellitus (T2DM) (**Table 2**). A metagenome-wide association study (MGWAS) in a large-scale population-based cohort study in China based on deep shotgun sequencing of the gut microbial DNA from 345 Chinese individuals. discovered a decreased trend in the abundance of *R. intestinalis* in T2DM patients (Qin et al., 2012). These results were confirmed in subsequent studies (Tilg and Moschen, 2014). However, the big question is whether different types of diabetes determine the diversity of the gut *Roseburia* population. Another investigation that evaluated the gut microbiota and metabolism in patients with type 1 diabetes mellitus (T1DM) reported lower *R. intestinalis* abundance (Leiva-Gea et al., 2018). Furthermore, a systemic review involving 26 studies, including 2,600 children and 189 adults, revealed that the most significant bacterial alterations in T1DM patients involved *Roseburia* spp. (Jamshidi et al., 2019). Taken together, these findings coincide with the essential role of butyrate in glucose homeostasis. Meanwhile, obesity shares the same difference in gut commensal compositions. A study by (Tims et al., 2013) recruited 40 monozygotic twin pairs and explored the association of microbial signatures with body mass index (BMI). The results revealed the positive correlation between *R. intestinalis* and BMI; that is, individuals with higher BMI carried more abundant butyrate producers. Another study reported that children and adolescents with primary hyperlipidemia had a lower abundance of *Roseburia*, suggesting the correlation of the *Roseburia* genus with the changes in lipidemic parameters (Gargari et al., 2018). These pieces of research demonstrate that *R. intestinalis* plays an extensively instrumental role in metabolic disorders. However, further studies are warranted to elucidate whether *R. intestinalis* plays a more pivotal role in metabolic diseases than do widely studied microorganisms, such as *Akkermansia muciniphila* and Christensenellaceae. Some of the significant shortcomings of metabolism research include the lack of an effective design for primary bacterial culture, identification of animal and cell experimental function, and necessary clinical trials. Perhaps, *R. intestinalis* will be included in mixed probiotic prescriptions for metabolic disorders in the future.

## Nervous System

The microbiota–gut–brain axis modulates the interaction between the nervous system and the intestinal environment. Bacterial composition and bacterial metabolites influence the function of both the enterocytes and exocrine glands. The effects of exocrine molecules on the enteric nervous system are either direct or indirect. Compelling evidence shows that immune cells such as ILC3 can interact with the colonic glia to form a glia–ILC3 axis, whereas microbes, including *Roseburia*, transmit signals to the colonic glia to stimulate the IL-22 production of ILC3 mediated by glial neurotrophic factors (Bernink et al., 2016; Ibiza et al., 2016). These complicated interaction axes involve microbes, immunity, and nerve. Therefore, gut bacteria such as *Roseburia* exert a potential impact on the pathogenetic process of nervous system diseases (**Table 2**). An investigation by Prof. Wei demonstrated that *R. intestinalis* influenced the 5-HT level and GFAP expression in colonic tissue, alleviating the depression-like behavior in mice (Xu et al., 2021). This piece of evidence supports the role of *Roseburia* in neural disorders. In another study, the mucosal and fecal 16S rRNA-based assay of patients with Parkinson’s disease (PD) and controls showed that *Roseburia* was significantly more abundant in controls than in PD patients (Keshavarzian et al., 2015). Additionally, when the fecal microbiota obtained from PD patients or healthy controls were transplanted into germ-free mice *via* oral gavage, the results indicated that the abundance of *Roseburia* spp. increased in animals colonized with microbiota from PD patients (Keshavarzian et al., 2015). It was speculated that *Roseburia* spp.-associated gut dysbiosis promoted the aggregation of α-synuclein *via* colonic motor defect, and the subsequent specific changes enhanced neuroinflammation and the progression of PD (Sampson et al., 2016). Considering the impact of the transplant on the actual dysbiosis of PD patients, the divergent results should be analyzed carefully and cautiously. Aside from PD, depression is another global neurological concern. Previous evidence indicated a lower abundance of *Roseburia* in major depressive disorder (MDD) patients than in the matched healthy group (Zheng et al., 2016). In the same study, fecal microbiota transplantation (FMT) from MDD patients to germ-free recipient mice could induce depression-like behaviors. Moreover, the transplanted depression mice were characterized by an enriched carbohydrate and amino acid metabolism (Zheng et al., 2016). Nevertheless, cohort data from a study by Jiang et al. revealed a relatively more abundant *Roseburia* in active MDD patients than in the healthy group (Jiang et al., 2015). Liu et al. confirmed that the reduced *Roseburia* abundance could be an independent label for MDD compared to diarrhea-predominant IBS (D-IBS) (Liu et al., 2016). Therefore, the specific microbial profile of nervous disorders should be defined and clarified cautiously in consideration of the differences in the nationalities of the subjects, control baselines, fecal storage, digestive tract problems, and the detection methods used. With FMT, one can partly prove the primary function of gut *Roseburia*. However, interactions between bacteria and neurons are more complex than are other interactions such as bacteria–epithelium or bacteria–immunity; mechanism studies are lacking. The advancement in the culture techniques of the human microbiota and culturomics has provided a platform to elucidate and uncover new details on the explicit role of *Roseburia* in neural disorders.

## Circulation and Hematology

Metabolites derived from *R. intestinalis* can infiltrate the gut barrier and circulate in the artery and the vein. As such, it is imperative to investigate the effects of *R. intestinalis* in circulatory diseases (**Table 2**). A classic role of *Roseburia* has been proven strongly in the modulation of atherogenesis. In a Swedish study, patients with symptomatic atherosclerosis had less abundant *Roseburia* than did healthy subjects (Karlsson et al., 2012). Moreover, gut metagenomes are enriched in genes encoding metabolic pathways forming atherosclerotic plaques. Kasahara et al. revealed a negative correlation of *Roseburia* spp. with the development of atherosclerotic plaques in a mouse model. The study established a murine model colonized with *R. intestinalis* and fed the transplanted gnotobiotic mice with a high concentration of plant polysaccharides (Kasahara et al., 2018). The results showed that *R. intestinalis* contributed to the reprogramming of metabolism from glycolysis to fatty acid utilization and the reduction of systemic inflammation. In addition, *R. intestinalis* inhibited atherosclerotic plaque formation and ameliorated atherosclerosis (Kasahara et al., 2018). A similar phenomenon had been observed in coronary artery disease (CAD) reported by two published large-scale population-based studies by Liu et al., who recruited 161 CAD patients and 40 healthy controls, and Zhu et al., who enrolled 70 CAD patients and 98 healthy controls. These two studies characterized bacterial co-abundance group changes at different disease stages and demonstrated a significant depletion of *Roseburia* in the CAD group (Liu et al., 2019; Zhu, Q. et al., 2018). In another research group, MGWAS analysis showed a lower abundance of *Roseburia* spp. in hypertension patients than in healthy controls (Yan et al., 2017). A related animal study also reported reduced *Roseburia* abundance in a two-kidney-one-clip (2K1C) hypertensive rat model (Qin et al., 2018). Furthermore, Zheng et al. demonstrated a disrupted abundance of bacteria, including *Roseburia*, in experimental rats from a 4-week heart failure rat model (Zheng et al., 2019). Collectively, these findings suggest a close correlation of the diseases of the circulatory system with *Roseburia*.

Regarding hematology, studies on disease microbiome are scanty. In one of the investigations, decreased *Roseburia* abundance was detected in the fecal samples of lymphoblastic leukemia patients (Rajagopala et al., 2016). Another study revealed that mice fed with pectic oligosaccharides had increased intestinal *Roseburia* spp., and the diet ameliorated leukemia progression in a leukemia mouse model (Bindels et al., 2015). These findings may unravel the role of *Roseburia* in leukemia. Moreover, studies conducted in Peru and Switzerland reported that iron-deficient anemia patients had a lower fecal *Roseburia* abundance than did the controls (Dostal et al., 2012; McClorry et al., 2018). These findings provide an insight into the interaction between hematology and microbiology. In this view, we believe that *R. intestinalis* sustains the stability of the gut ecology and provides persistent indirect benefits in circulation and hematology; the effect is slow, but persistent. It is undeniable that multiomics and single-cell sequencing technologies have greatly improved the breadth and depth of bacteria–circulation research. These new technologies will guide the examination of circulation and the blood effects of germ-free animals after *R. intestinalis* intervention.

## Infection and Cancer

Microbiota-reactive CD4^+^ T cells are essential in intestinal homeostasis because they produce permeability-sustaining cytokines and provide a large pool of T-cell alternatives that repel pathogens (Hegazy et al., 2017). Human immunodeficiency virus (HIV) impacts vast global populations, posing heavy public burdens and life-threatening risks. Evidence shows that HIV attacks the immune system (including the enteric defending barrier) whereby it infects and depletes CD4^+^ T cells. The consequently low CD4^+^ T cells induce gut dysbiosis, systemic inflammation, and clinical complications in chronic HIV patients. Researchers have detected low *R. intestinalis* abundance in the colonic mucosa of HIV patients compared to that in uninfected patients. In a recent investigation, the abundance of *R. intestinalis* was found to be inversely correlated with bacterial translocation, immune activation, and vascular inflammation (Dillon et al., 2017). Of note is that data from rectal mucosal microbiota rather than feces indicated depleted *Roseburia* in HIV patients who did not receive antiretroviral therapy (McHardy et al., 2013). However, the mechanism of dysbiosis induced by low CD4^+^ T cells is complex. In feces of HIV patients, low microbial gene counts (LGCs) but enrichment in *R. intestinalis* correlated with low nadir CD4^+^ T-cell counts, which could predict gut dysbiosis in HIV (Guillen et al., 2019). In addition, a significantly enriched *Roseburia* abundance was reported in active tuberculosis (TB) patients, and *Roseburia* promoted TB pathophysiology by enhancing the anti-inflammatory milieu in the host (Maji et al., 2018). However, a contrary report of a Chinese trial confirmed a decrease in *Roseburia* abundance in TB patients (Hu et al., 2019). Therefore, further investigation is warranted to address these divergent results.

The cancer-associated microbiome has always been a preference in cancer research. It is worth noting that the microbiota contributes to carcinogenesis and tumor progression in colorectal cancer (CRC), hepatocellular carcinoma, pancreatic tumor, and cervical cancer. Therefore, the microbiota can serve as early diagnostic biomarkers to guide precise cancer treatment. A previous investigation by Liang et al. (2017) reported that *R. intestinalis* abundance was decreased in CRC patients compared to healthy controls. Notably, the change in *R. intestinalis* abundance is consistently significant among cancer-associated microbiome studies. In a study by Yu et al. using MGWAS, the abundances of some marked microbes, including *R. intestinalis*, were decreased in 74 Chinese patients with CRC compared to the controls. These results were consistent with validated data from cohorts in Denmark, France, and Austria (Yu et al., 2017). Similar findings were also documented in independent studies (Wang et al., 2012; Chen et al., 2013). After a careful evaluation of the literature reports on CRC microbiota based on their large size and consistent results, we provide evidence that *R. intestinalis* may be a definite protective factor and a reliable biomarker in CRC. However, for other cancers (nasopharyngeal carcinoma and cervical cancer), divergent results were found (Jiang et al., 2019; Wang et al., 2019). It is notable that both studies displayed connections between *Roseburia* abundance and cancer risk, although the results were not related to the beneficial effect of *Roseburia*. Generally, cancer-associated microbiota is relevant to the cancer microenvironment. *R. intestinalis* may contribute to the dysfunction of the immune microenvironment and metabolic reprogramming of cancer cells. Recent evidence demonstrated an association of the Clostridiales members of the gut microbiota with a lower tumor burden in mouse models of CRC. Notably, these commensal species, including *R. intestinalis*, were also significantly reduced in CRC patients compared to healthy controls (Montalban-Arques et al., 2021). A mix of four Clostridiales strains (CC4) in mice prevented and successfully cured CRC as a stand-alone treatment in anti-PD-1 therapy. Also, the bacterial treatment induced a high infiltration of CD8^+^ T cells within the tumor, making them more immunogenic (Montalban-Arques et al., 2021). A single application of *R. intestinalis* showed higher efficacy than that of CC4 (Montalban-Arques et al., 2021). This section has shown a potential synergy between tumor immunotherapy and microbes. Thus, further cancer-associated microbial studies are warranted to elucidate the role of *R. intestinalis* in tumor immunotherapy. Although metagenome results could not distinguish the above role, germ-free animals and primary bacterial culture of tumors have great application prospects.

## Respiratory, Nephrology, and Others

Acute respiratory distress syndrome (ARDS) is an acute lung injury that occurs mostly following an infection, chemical airway burn, or other factors. ARDS is characterized by a disruption of the alveolar epithelial and endothelial barrier, inflammatory effusion, ventilation defect, and severe respiratory failure. Intestinal microbial dysbiosis may aggravate ARDS. A previous study conducted using an acute lung injury rat model (rats were intratracheally instilled with lipopolysaccharides) demonstrated a higher diversity index and a lower number of *Roseburia* spp. in the gut flora of model rats compared to that of the control group (Li et al., 2014).

In most cases, chronic kidney disease (CKD) is comorbid with metabolic and cardiovascular complications, and most patients can progress into end-stage renal disease. In addition, a deteriorated internal environment frequently accompanies an impaired gut barrier and low-grade inflammation. CKD patients have been shown to harbor shrunken percentages of beneficial microbes, including *Roseburia* (Xu et al., 2017). In another study, a reduced abundance of *Roseburia* was reported in the end-stage renal disease group and was negatively correlated with C-reactive protein and cystatin C (Jiang et al., 2017). Some studies have shown a negative correlation of *Roseburia* with CKD severity at different stages of the disease (Wu, I. W. et al., 2020), and related studies continue to emerge. This review has repeatedly confirmed the beneficial role of *Roseburia* in different diseases. However, the precise role of *Roseburia* spp. could hardly be defined because dysbiosis is expected in such diseases. Nevertheless, we are more convinced that *Roseburia* is a potential probiotic.

## *R. intestinalis* as a Probiotic Candidate: The Roads to Explore

**Figure 3:**
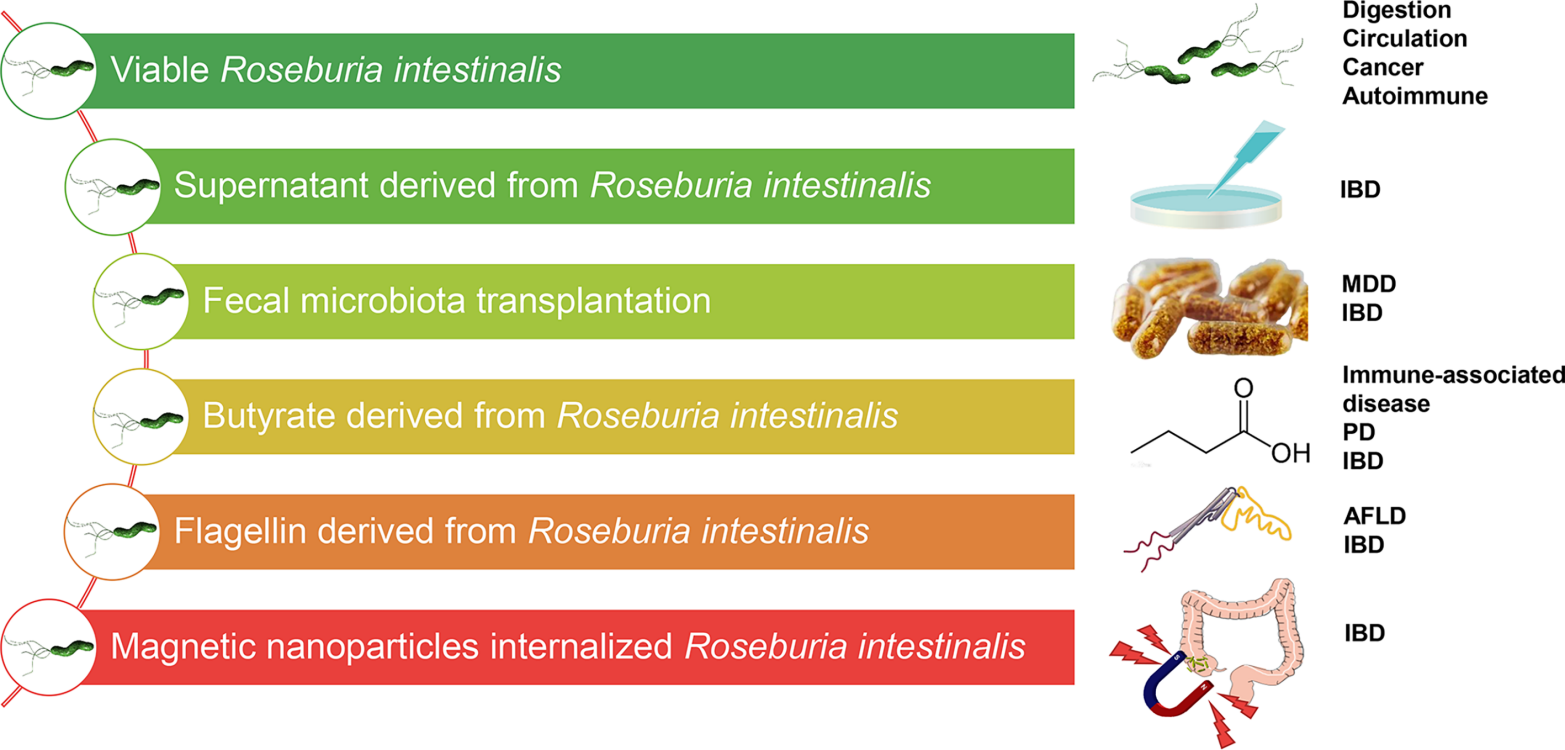
Different therapeutic methods targeting *Roseburia intestinalis* in potential diseases. *IBD*, inflammatory bowel disease; *MDD*, major depressive disorder; *PD*, Parkinson’s disease; *AFLD*, alcoholic fatty liver disease.

Many clinical studies of human cohorts using established disease models have documented the protective role of *Roseburia* spp., especially *R. intestinalis*. Nearly all the data demonstrate a probiotic role of *R. intestinalis* in preventing infection and in relieving and reversing the pathological process. Notably, *R. intestinalis* has displayed a beneficial role in different modes, such as butyrate, supernatant, flagellin, and organism effect. In combination with other emerging evidence, findings from our research group have revealed the effect of *R. intestinalis* on Tregs, ILC3, Th17, and macrophages and the stimulation of anti-inflammatory cytokines (IL-22, IL-17, IFNγ, TSLP, and OSM). There is evidence that different technologies have been applied to modulate *R. intestinalis*-associated disease treatment (**Figure 3**), and the significance of FMT as a microbial treatment choice in diseases is well highlighted. For example, *Clostridium difficile* colitis is associated with microbiota dysbiosis caused by the overuse of antibiotics. FMT, as standard therapy, reverses microbiota dysbiosis and maintains the natural microbiota. FMT also demonstrates favorable potential in managing chronic inflammatory disorders, including UC. A randomized, double-blinded controlled trial, for instance, demonstrated that the application of FMT to manage active UC patients improved the microbial diversity and altered the bacterial taxa. Furthermore, patients in the remission group displayed more abundant *Roseburia* spp. and higher SCFA levels than those who experienced no remission after FMT (Paramsothy et al., 2019). Another investigation proposed that *Roseburia* species could be the prominent candidates for the probiotic treatment of patients with IBD based on the consistent results in the experimental center (Kellermayer, 2019). In our previous study (Yang et al., 2020), CD patients who received FMT showed significant improvement in the number of operational taxonomic units (OTUs) and Shannon diversity index than did donors, 2 weeks after FMT, which included *R. intestinalis*. Collectively, *R. intestinalis* might play a beneficial role in the recovery of the gut microenvironment and the improvement of disease pathophysiology through FMT.

It is well known that the butyrate produced by *R. intestinalis* is a prebiotic. Researchers have attempted to directly or indirectly administer butyrate or butyrate “carriers” such as tributyrin (Wächtershäuser and Stein, 2000; Tuleu et al., 2001; McNabney and Henagan, 2017). For effective absorption, rectal butyrate enema had been previously introduced in UC treatment (Hove et al., 1995). Similarly, our research group revealed the therapeutic effect of *R. intestinalis* by suspension gavage in mice. In this view, we strongly suggest a need to develop viable probiotics based on *R. intestinalis* in medicine. Whole-grain diets with abundant fiber can also be fermented using *R. intestinalis*. Of note is that other special prebiotic supplements, such as acetate, xylan, β-mannans, and protein hREG3α, alter the abundance of *R. intestinalis* and butyrate production and promote niche superiority. As such, the application of *R. intestinalis* can benefit from advancement in the development of materials. Recently, in our laboratory, we developed a magnetic iron oxide nanoparticle (MION) internalized *R. intestinalis*, and the magnetic field can be directed both *in vitro* and *in vivo* (Xiao et al., 2019). Additionally, a careful investigation of the viability and cytotoxicity of MION internalized *R. intestinalis* and a custom-made magnetic guide facility were conducted *in vitro*. The results demonstrated good orienteering and the anti-inflammatory effect of MIONs in the TNBS-induced colitis mouse model (Xiao et al., 2019).

More findings from our laboratory indicated that the flagellin from *R. intestinalis* displayed a favorable anti-inflammatory effect *via* intraperitoneal injection (Quan et al., 2018; Wu, X. et al., 2020). These results strongly suggest that purified flagellin may be a safe and effective therapy for intestinal immune modulation. Furthermore, some tryptophan metabolites, including indoles, may be a new choice targeting *R. intestinalis*-associated mechanisms, for example, IL-22 production and ILC3 activation by the AhR. Therefore, further well-designed studies and credible clinical trials should be conducted to clarify the beneficial effects of *R. intestinalis*.

Regarding the safety of *R. intestinalis*, our laboratory is evaluating its risk in mouse symptoms, circulating system, and organs systemically. Further data will estimate its safety. Evidence should be accumulated in its safety and proper uptake dose. As a probiotic candidate, preclinical evaluation and clinical trials are urgently needed to prove its therapeutic potential in diseases.

## Discussion

In 2014, the International Scientific Association for Probiotics and Prebiotics consensus statement updated the scope and appropriate use of the term probiotic. The reinforced concept defines probiotics as live microorganisms that, when administered in adequate amounts, confer a health benefit to the host. The expert consensus listed several probiotic candidates, including *A. muciniphila*, *F. prausnitzii*, *Roseburia* spp., and *Eubacterium hallii* (Hill et al., 2014). It is expected that therapeutic approaches targeting *R. intestinalis* will significantly improve specific human pathogenic status. Notably, inflammatory bowel disease exhibits significant changes in the abundance of *R. intestinalis* and benefits the most from its modulations. Recent breakthroughs also suggest the probiotic role of *R. intestinalis*, which has a widespread effect in preventing intestinal inflammation and maintaining energy homeostasis based on its metabolites, and noumenon. *R. intestinalis* could impact the functions of immune cells and the release of cytokines through its metabolites, butyrate and flagellin, and other unknown supernatant components. Advancements in primary culture technology, culture omics, single-cell sequencing, and metabonomics technology are expected to help researchers in elucidating the role of *Roseburia* spp. in order to lay a solid foundation in the maintenance of human health and treatment strategies for diseases in the future. Based on the available evidence, focused, well-designed randomized controlled trials, and future high-quality systematic reviews and meta-analyses should be urgently conducted to uncover the probiotic role of *R. intestinalis*. In conclusion, as a significant butyrate producer, *R. intestinalis*, which belongs to the *Roseburia* species, has great application prospects in the development of novel probiotics. It is possible that *R. intestinalis* can exert a specific and a significant therapeutic influence in different diseases associated with microbial dysbiosis.

## Author Contributions

KN reviewed all the literature and wrote the manuscript. KM, WL, ZS, ZY, TT, and XM undertook most of the group’s research work and published their findings. They also discussed each part of the manuscript and helped form our viewpoints. YY funded us and edit the manuscript. XW conducted the research group including above authors and edited the manuscript. All authors contributed to the article and approved the submitted version.

## Funding

This project was supported by the National Natural Science Foundation of China (NSFC nos. 81970494 and 81800500) 81970494 was grant to XW and 81800500 to YY.

## Conflict of Interest

The authors declare that the research was conducted in the absence of any commercial or financial relationships that could be construed as a potential conflict of interest.

## Publisher’s Note

All claims expressed in this article are solely those of the authors and do not necessarily represent those of their affiliated organizations, or those of the publisher, the editors and the reviewers. Any product that may be evaluated in this article, or claim that may be made by its manufacturer, is not guaranteed or endorsed by the publisher.

## Abbreviations

IBD, inflammatory bowel diseases; UC, ulcerative colitis; IBS, irritable bowel syndrome; GPCRs, G-protein coupled receptors; TSLP, thymic stromal lymphopoietin; GLP-1, glucagon-like peptide-1; PYY, peptide YY; OSM, oncostatin M; hREG3α, human regenerating family member 3 alpha; TLR5, Toll-like receptor 5; IDO1, indoleamine2,3-dioxygenase-1; HIF1A-AS2, lncRNA (HIF1A-AS2); FISH, fluorescence *in situ* hybridization; MGWAS, metagenome-wide association study; NAFLD, non-alcoholic fatty liver disease; AFLD, alcoholic fatty liver disease; CRD, chronic rheumatoid disease; APS, antiphospholipid syndrome; CAD, coronary artery disease; HIV, human immunodeficiency virus; CRC, colorectal cancer; ARDS, acute respiratory distress syndrome.
